# Functional and Radiological Results Following Revision Blade Plating and Cephalomedullary Nailing in Aseptic Trochanteric and Subtrochanteric Nonunion

**DOI:** 10.3390/jcm13123591

**Published:** 2024-06-19

**Authors:** Julia Rehme-Röhrl, Andreas Brand, Annika Dolt, Dag Grünewald, Reinhard Hoffmann, Fabian Stuby, Uwe Schweigkofler, Christian von Rüden

**Affiliations:** 1 Department of Trauma Surgery, BG Unfallklinik Murnau, 82418 Murnau, Germany; 2 Institute for Biomechanics, Paracelsus Medical University, 5020 Salzburg, Austria; 3 Institute for Biomechanics, BG Unfallklinik Murnau, 82418 Murnau, Germany; 4 Department of Trauma and Orthopedic Surgery, BG Unfallklinik Frankfurt, 60389 Frankfurt, Germany; 5 Department of Trauma Surgery, Orthopaedics and Hand Surgery, Weiden Medical Center, 92637 Weiden, Germany

**Keywords:** aseptic nonunion, Harris Hip Score, intramedullary nail, outcome, proximal femur fracture, SF-12, 95-degree angled blade plate

## Abstract

**Background:** Trochanteric and subtrochanteric fractures result in nonunion in more than 20% of cases. The aim of this study was to assess the functional and radiological results following revision cephalomedullary nailing and 95-degree angled blade plating in aseptic trochanteric and subtrochanteric nonunion. **Methods:** In a retrospective multi-center study between January 2010 and December 2020, a total of 68 consecutive patients (21 women and 47 men) from two European level I trauma centers with the diagnosis of aseptic nonunion were recruited. Follow-up assessment and the patients’ convenience were assessed using the Harris Hip Score, Visual Analog Scale for pain at rest and on stress/exertion and Short Form-12. **Results:** The patients’ mean age was 57 (range 26–85) years. After a follow-up period of 12 months, one case of persistent nonunion in the cephalomedullary nail group and 10 cases in the blade plate group were identified. The mean duration of surgery was 137 ± 47 min in the cephalomedullary nail group and 202 ± 59 min in the blade plate group (<0.0001). Short-term postoperative complications included wound dehiscence, bleeding, mismatched screw and hematoma. The mid-term results 12 months after surgical revision demonstrated significantly different osseous union rates (*p* = 0.018). The long-term functional outcome according to the Harris Hip Score 6 years (range 2–10) after revision surgery demonstrated 81 ± 21 points in the cephalomedullary nail group and 64 ± 23 points in the plate group (*p* = 0.026). **Conclusions:** This study demonstrated that the revision treatment of trochanteric and subtrochanteric nonunion using a 95-degree blade plate or cephalomedullary nail resulted in a high percentage of osseous union, with a low incidence of complications and good functional results for both methods.

## 1. Introduction

Various fixation procedures are currently established in trochanteric and subtrochanteric fracture treatment, whereby intramedullary nailing appears to be the primary treatment option. In primary care, extramedullary implants such as the dynamic hip screw (DHS) are increasingly less important. In up to 20% of trochanteric and subtrochanteric fractures, there is no osseous healing, and thus, the symptomatic nonunion is associated with significant limitations and pain for those patients affected [1]. Potential causes for nonunion development are usually related to improperly respected biomechanics, initial implant placement or implant selection, excessive remaining bony gaps in the fracture area or infection [2,3]. Varus and retroversion deviation as well as the loss of the medial abutment have an unfavorable effect on fracture healing [4]. Despite the implant-typical biomechanical advantages of intramedullary force carriers, such as the shortening of the lever arm towards the load axis, various complications such as implant dislocation, implant fracture or a secondary loss of reduction are reported [5]. Recently, biological factors such as the extent of the soft tissue dissection during proximal femoral surgery and the potential implications of the sterile inflammatory response magnitude in terms of the invasiveness of the reduction technique used in trochanteric and subtrochanteric fracture internal fixation have come into focus [6]. In addition, patient-related risk factors include nicotine abuse, obesity, diabetes mellitus and the use of certain medications [6]. Moreover, especially in elderly patients, general risk factors such as clinical preconditions, age and management in the pre- and postoperative in-hospital course are also of importance for the development of fracture union and clinical outcomes [7]. Especially in the case of implant failure, re-osteosynthesis is known to be the gold standard, but there is still no common consensus for the standard surgical treatment concept for aseptic trochanteric and subtrochanteric nonunion. Several studies have proposed either intramedullary nailing or extramedullary revision procedures [4,8,9].

Therefore, the focus of this clinical study was to compare the surgical revision tools 95-degree angled blade plate and cephalomedullary (CM) nail in terms of application data, complication rates, healing rates and functional and radiological results, aiming to evaluate the advantages and disadvantages and to highlight the superiorities of both procedures.

## 2. Patients and Methods

**Figure 1:**
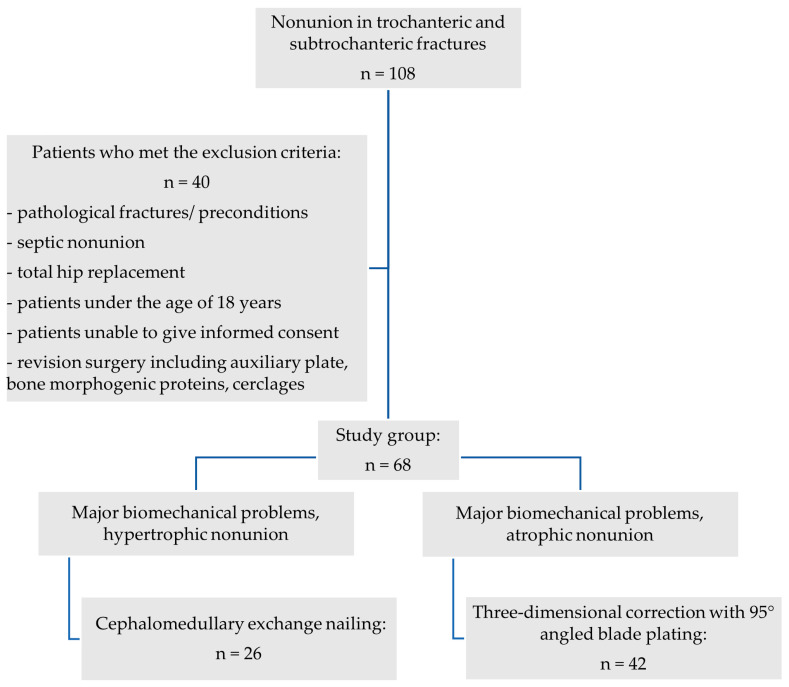
Overview of patients’ inclusion and therapeutic decision-making process.

**Figure 2:**
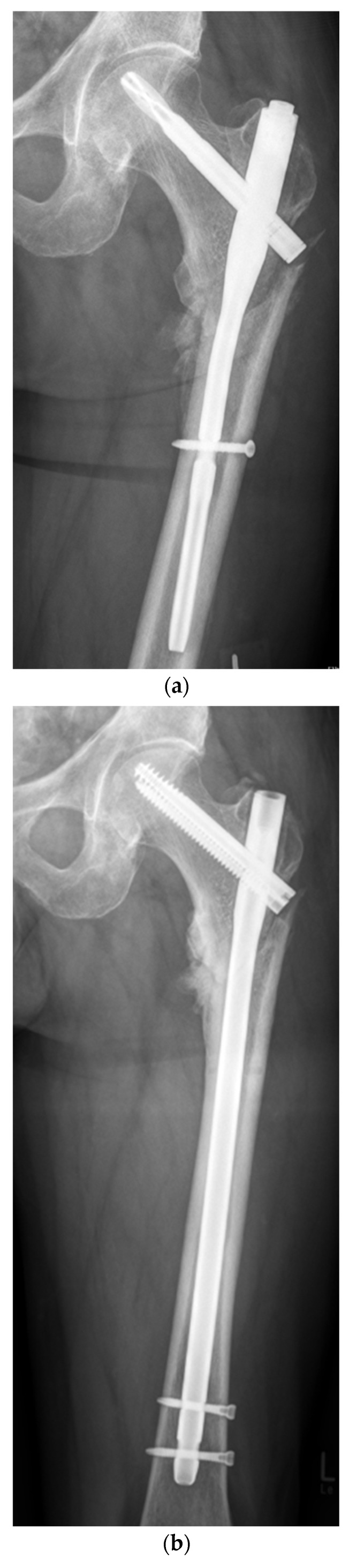

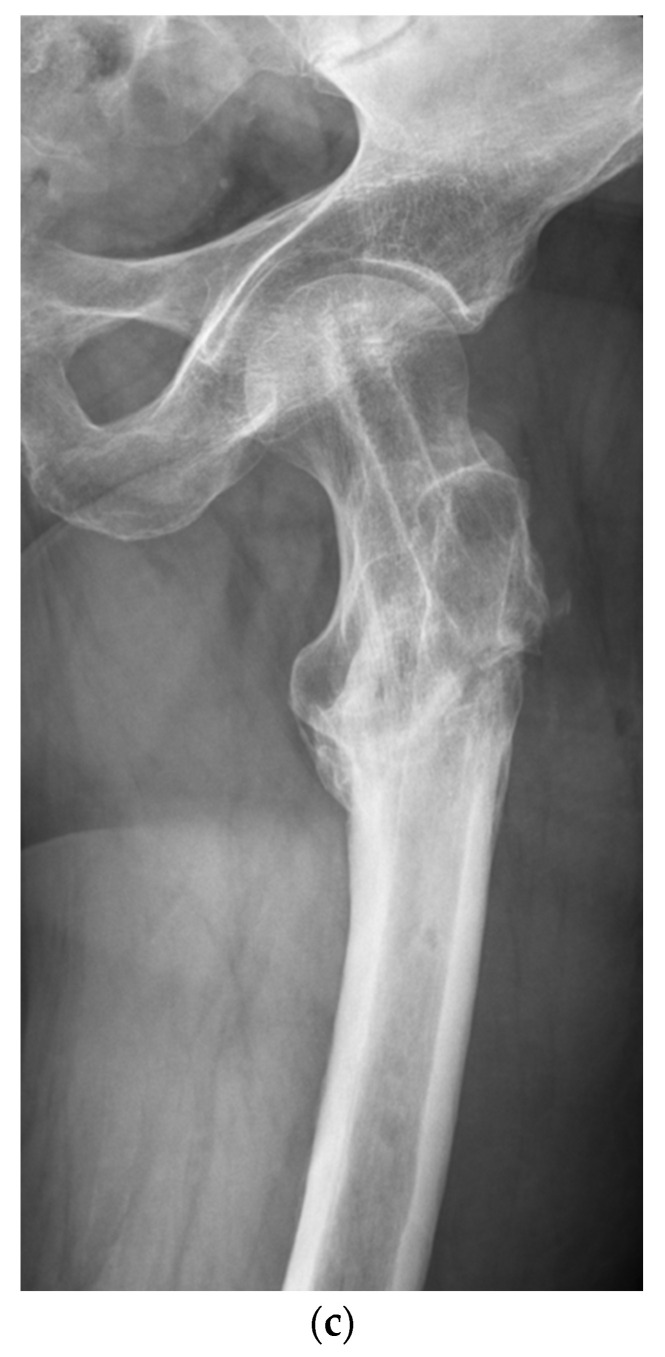
(**a**) Aseptic nonunion 9 months following cephalomedullary nail fixation in a multi-fragmentary subtrochanteric fracture in a 58-year-old male patient. (**b**) Osseous healing three months after revision surgery using a 360 mm × 13 mm Intertan™ cephalomedullary nail. (**c**) Situation after metal removal 36 months following surgical revision.

**Figure 3:**
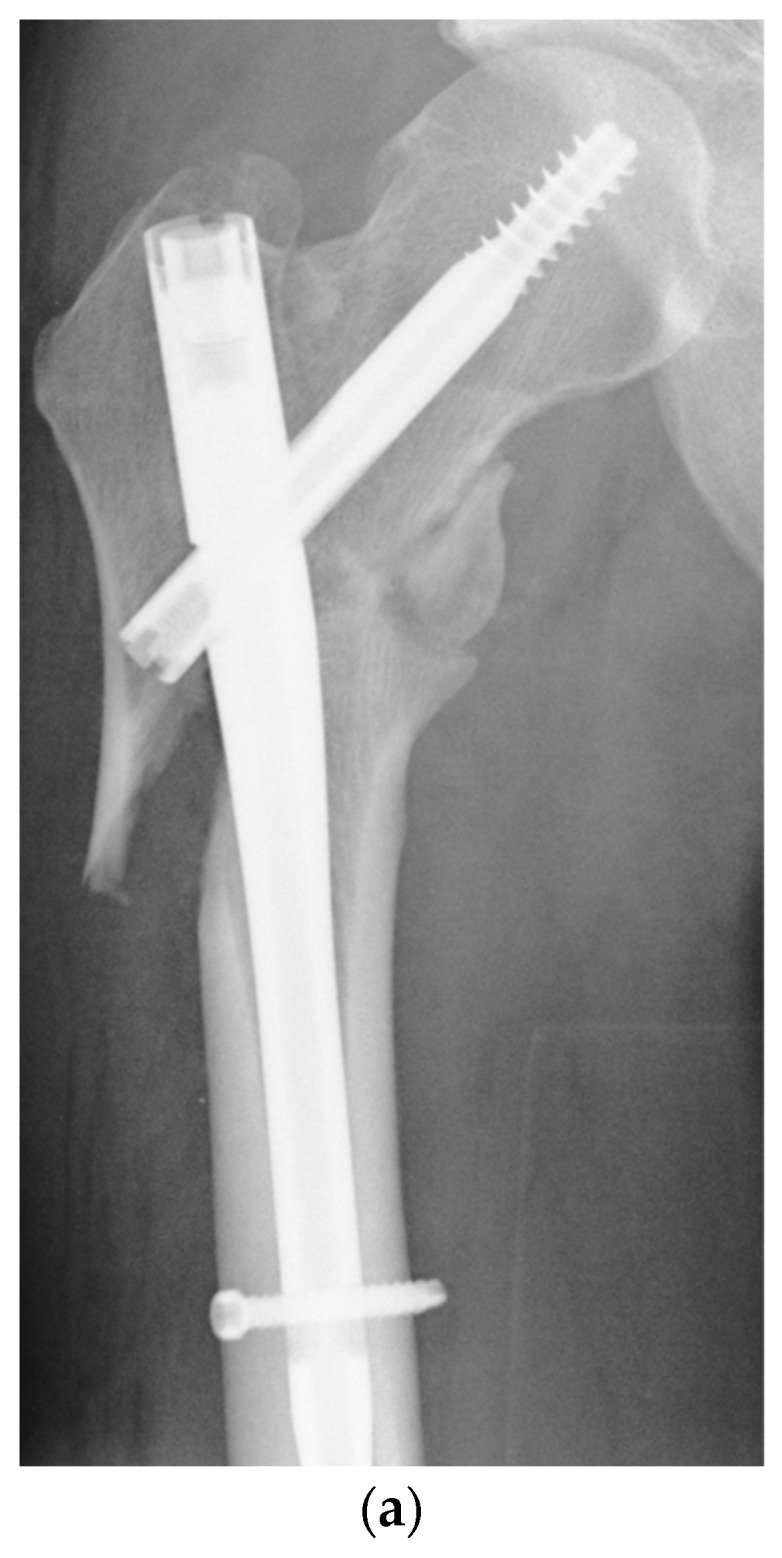

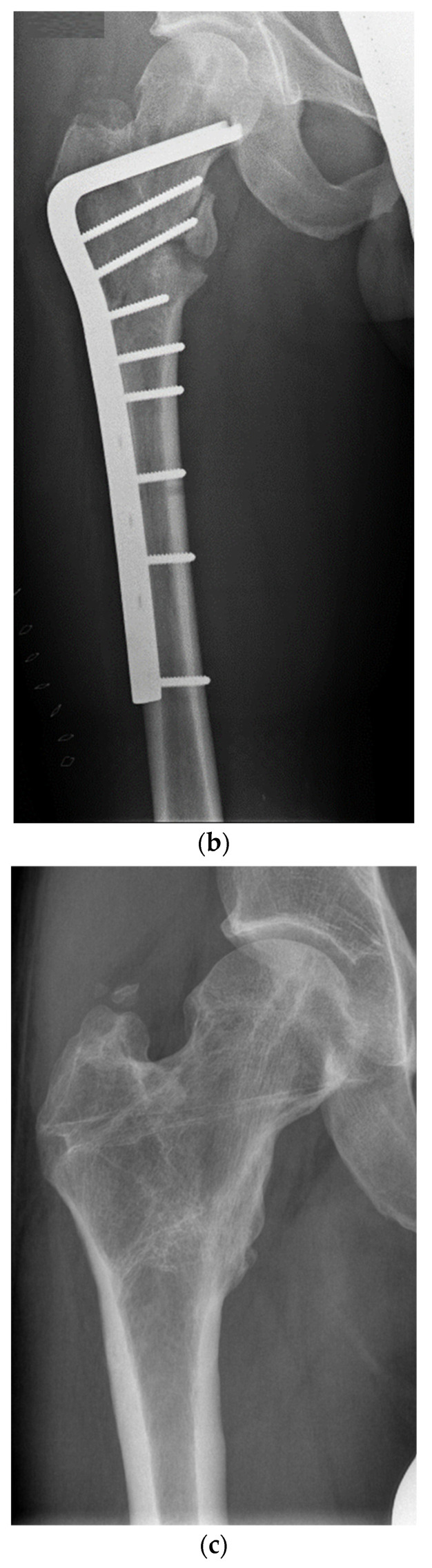
(**a**) Aseptic nonunion 6 months following cephalomedullary nail fixation in a reversed multi-fragmentary trochanteric fracture in a 48-year-old male patient. (**b**) Osseous healing three months after revision surgery using a 95-degree blade plate. (**c**) Situation after metal removal 12 months after surgical revision.

A retrospective analysis of the in-house databases of two European level I trauma centers was carried out for consecutive patients from January 2010 to March 2020. A total of 68 consecutive patients (21 women and 47 men) with the diagnosis of aseptic nonunion following AO/OTA 31 A1, 31 A2, 31 A3, very proximal 32 A13, 32 B13 and 32 C13 fractures were included (Figure 1; *p* < 0.0001). Patients aged 18 years and older who had undergone surgical revision for trochanteric and subtrochanteric nonunion with revision cephalomedullary nailing (Gamma3^®^, Stryker Corp., Kalamazoo, MI, USA; Trigen™ Intertan™, Smith & Nephew Inc.; Memphis, TN, USA; Figure 2a–c) and 95-degree angled blade plating (DePuy Synthes GmbH, Oberdorf, Switzerland; Figure 3a–c) were included. Pathologic fractures, fractures treated by total hip arthroplasty (THA) and periprosthetic fractures or extramedullary fixation devices were excluded from the study a priori, as were patients younger than 18 years and patients unable to give consent. The inclusion and exclusion criteria were strictly controlled and respected.

### 2.1. Diagnostic Work-Up

Only cases with complete preoperative medical reports, biplanar radiographs and/or CT scans and intraoperative radiographs were analyzed.

Patients with clinical signs of local infection, positive blood samples including increased C-reactive protein and white blood cell values and patients with any evidence of infection in the anamnesis were directly excluded from the study. Aseptic nonunion was defined as a fracture that will not heal without further medical intervention regardless of the duration of treatment [10]. The initial fracture type was classified by the same two senior orthopedic surgeons. The AO/OTA classification was used [11].

The caput-collum-diaphyseal (CCD) angle was compared preoperatively with the opposite side. If it was less than −10°, the indication for valgization using a 95-degree angled blade plate was given; if not, replacement with cephalomedullary exchange nailing was performed. Additional factors such as previous diseases, soft tissue situation and bone quality were included in the indication.

### 2.2. Surgical Procedures

The surgical procedures were performed by six different experienced senior surgeons familiar with the methodology and implants. All surgical procedures were carried out in a standardized manner according to manufacturers’ recommendation in the supine or side position through a standard lateral approach. Additional cerclage wires, auxiliary plates, bone morphogenetic proteins (BMPs) or bone grafts were not used in any case. In all cases, the original nail was removed prior to the insertion of the revision implant in the same surgical intervention. The revision procedure was minimally invasive (estimated length of incision of 4–5 cm) and generally allowed for the early postoperative mobilization of the patient. The aim of the treatment was to achieve biomechanical stability using a longer nail with a larger diameter of at least 2 mm than the original nail. However, this was only possible if the initial varus–valgus deviation and the three-dimensional hip angle could be reduced anatomically prior to the insertion of the revision cephalomedullary nail and if it was not identified to be the reason for biomechanical instability [12]. If this occurred, a combination of biological revision and mechanical improvement might be performed, whereby a complex corrective osteotomy with the aim of valgization in the proximal femur should be considered using a 95-degree angled blade plate. This surgical concept included an open procedure with a larger estimated length of incision of 10–20 cm, depending on the length of the plate, and a higher level of invasiveness due to the osteotomy and the reduction in bone fragments in three-dimensionally correct anatomical positions.

### 2.3. Outcome Measures

The databases were evaluated regarding the initial medical history, medical treatment and the healing process of the primary and secondary surgical procedures. In addition, patient-specific factors such as age, gender, body mass index (BMI), concomitant diseases and application data (duration of surgery, duration of inpatient stay) and contemporary complications (hematoma, seroma, blood transfusion) were observed. Functional follow-up assessment and the patients’ convenience were examined using the Harris Hip Score (HHS) [13], the Visual Analog Scale (VAS) for pain at rest and on stress/exertion and the Short Form (SF)-12 [14] to assess the health-related quality of life at different time points (fracture before treatment, as well as postoperatively and 3, 6, 9 and 12 months postoperatively). Radiological outcome was assessed by evaluating the initial implant position and the new implant position using the CCD angle in anterior–posterior and lateral radiographs. Osseous union was defined as the ability to bear full weight without pain, stability at the former nonunion site, bridging callus at four cortices or the absence of fracture lines [15].

### 2.4. Statistical Analysis

Statistical analysis was performed by one in-house independent statistician. The data were edited and analyzed using Excel^®^ 2016 for Windows^®^ (Microsoft Corp., Redmond, WA, USA). IBM SPSS^®^ Statistics Version 26 for Windows (IBM Corp., Armonk, NY, USA) was used for statistical analysis. Metric continuous data were tested for normality using the Shapiro–Wilk test. Depending on data normality, Student’s *t*-test or the Mann–Whitney test was used for statistical comparisons. Dichotomous variables related to complications, risk factors or preconditions were compared between both groups using the Chi-squared test. Comparisons of the AO/OTA fracture classification between groups were conducted using the post hoc Chi-squared test with Bonferroni correction. The level of significance was set at *p* < 0.05.

## 3. Results

An overview of all patients and their main basic data included in this study are displayed in Table 1. In 42 out of 68 patients, nonunion was corrected using the 95-degree angled blade plate. In the remaining 26 cases, a cephalomedullary nail was used. In terms of age, gender and days in the hospital, no statistical significance between both treatment groups could be observed. Due to indications, there was a naturally expected difference according to the AO/OTA classification between the treatment groups regarding 31A1 and 31A3 fractures (*p* < 0.0001).

Data concerning risk factors and pre-existing conditions in both treatment groups are provided in Table 2. There was no statistical significance in terms of these parameters between the treatment groups.

Short-term complications were assessed during the hospital stay with a focus on transfusion rates, local wound infection or the development of a hematoma (Table 3).

The length of the blade plate was evaluated in detail in terms of complication rates due to its broader surgical approach (Table 4). Five- and seven-hole blade plates were defined as “short” and 9- to 16-hole plates as “long”.

The mid-term complication rates demonstrated a statistically significant difference between both study groups due to the osseous union rates (Table 5).

**Figure 4:**
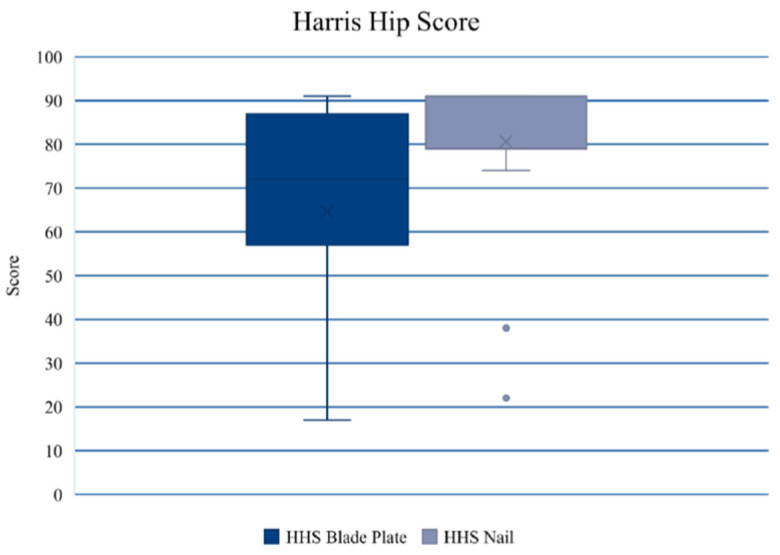
According to the HHS, significantly higher values were found in the CM nail group than in the blade plate group.

Follow-up assessment and the patients’ convenience with either the CM nail or 95-degree angled blade plate treatment were assessed using the Harris Hip Score (HSS; Figure 4), Visual Analog Scale (VAS) and Short Form (SF)-12. The median review period was 6 (range 2–10) years after revision surgery. An overview of the data from 43 patients (blade plate: 27; CM nail: 16) available for final follow-up is presented in Table 6 (loss of follow-up: 37%). Statistical significance was found in the HHS, with higher values in the nail group compared with the blade plate group.

## 4. Discussion

Fractures close to the hip are the most frequently treated fractures worldwide [16,17]. The mechanical failure of trochanteric and subtrochanteric fractures is an emerging complication due to their increasing prevalence [18]. A resulting nonunion is a challenging and rare situation. Delayed fracture healing or nonunion occurs in 10% of all bony fractures. This increases to over 30% in patients with corresponding risk factors [4]. The current study demonstrated even higher risk factor and precondition rates in both groups and therefore once again showed the importance of respecting the biology and biomechanical stability and anatomical reduction in the first place. Pre-existing preconditions such as smoking or alcohol abuse are also known as important risk factors for nonunion development [6]. However, these factors could not be influenced or reversed in the current study. Nevertheless, they always have to be taken into account during index surgery.

There are only a few studies dealing with the treatment of nonunion following proximal femoral fractures that highlight one revision procedure or another [19,20,21]. The choice of implant plays a decisive role during revision management. In a recent publication by Bhowmich et al., an algorithm for decision making in the management of proximal femoral nonunion based on the fracture pattern, anatomy, status of the bone healing and quality of the bone was suggested [22]. In the current study, the indication for the use of a CM nail or 95-degree angled blade plate was made according to the CCD angle and existing preconditions.

Biomechanically, the trochanteric and subtrochanteric nonunion observed in this study resulted from varus axis deviation during index surgery, which confirmed our own prior findings [23]. In cases where anatomic reduction cannot be achieved adequately, the biomechanical basis for the necessary intrinsic stability and subsequent bone healing is missing [24]. Therefore, anatomic reduction during index as well as revision surgery is of tremendous importance to increase the healing rate and to decrease the complication rate [25,26]. The medial column is under enormous loading pressure when the proximal femur is loaded axially. The lateral column, on the other hand, is under the influence of tensile forces [27].

In the case of trochanteric and subtrochanteric nonunion, there are basically two surgical options: minimally invasive reamed cephalomedullary exchange nailing or open revision followed by plate fixation. Both procedures may induce osseous healing and support biomechanics. In addition, in elderly patients, there is a third option for these situations: especially the most proximal ones might be treated by THA [28]. However, this treatment option was considered to be an exclusion criterion in the current study.

In a retrospective follow-up study of 50 diaphyseal femoral nonunion patients treated by reamed cephalomedullary exchange nailing using a longer nail with a larger diameter, Swanson et al. demonstrated that 100% osseous consolidation could be achieved over a period of 3 to 26 months [9]. Similar results could be demonstrated by Hak et al. and Shroeder et al. with healing rates of 78 and 86% respectively [29,30]. The current study supported these results as only one out of twenty-three patients did not heal following revision cephalomedullary exchange nailing. In this regard, several authors reported that revision surgery using cephalomedullary nailing might be a suitable treatment option for the aforementioned situations [31,32,33]. In comparison, cephalomedullary nailing is reported to combine superior biomechanical aspects with less invasiveness [19]. Compared with extramedullary devices, the nail is located closer to the vector of the force line, equalizing the shorter lever arm [34]. The only contraindication for its use might be seen in varus malalignment associated with a leak of compression on the bone fragments [35]. In this case, valgus correction and fixation with a 95-degree angled blade plate might represent a sufficient alternative. Therefore, the aim of this study was to evaluate potential differences between revision 95-degree blade plating and cephalomedullary nailing in aseptic trochanteric and subtrochanteric nonunion. The 95-degree angled blade plate was considered since the device respects all aspects of bone healing and biomechanics in three dimensions. However, the procedure is technically demanding and requires a relatively large surgical approach coupled with a longer operation time and higher risk of bleeding [8,9,36]. Whether these findings obtained in cadaveric bone might be applied to a clinical situation was examined in the current study. To the best of our knowledge, a direct comparison of the results of cephalomedullary exchange nailing and revision 95-degree blade plating in the long-term clinical course has not been performed yet.

Short-term complications were assessed in terms of the transfusion rate, local infection or necessary surgical revision. There were no differences observed between the treatment groups, which corresponds with the results provided by Vicenti et al. [21]. Thus, both procedures seem to be safe treatment options. Nevertheless, the duration of 95-degree blade plate surgery was significantly longer in our cohort. Wang et al. previously demonstrated that a prolonged surgery time was associated with increased postoperative delirium, especially in geriatric patients with a relevant spectrum of comorbidities [37,38]. In the current study, the length of the blade plate was also evaluated in detail in terms of complication rates due to its extended surgical approach. Concerning the blade plate length, we decided to define five- and seven-hole plates as “short” and nine holes and more as “long”. Astonishingly, there was no difference seen between the different plate sizes.

In contrast to earlier studies, the long-term results of the current study did not demonstrate any statistically significant difference regarding revision rates, but in terms of the osseous union rates, there was a difference at 12 months following revision surgery [21,33]. Interestingly, the complication rates were significantly higher in the blade plate group, especially related to surgical revision. This might be explained by the higher invasiveness of this surgical method. Unfortunately, due to the lack of complete datasets 12 months postoperatively, the further clinical course could not be observed.

One strength of this study might be seen in the relatively long follow-up period of 6 years after surgery on average. Despite this long period, the loss of follow-up was only 37%. Patients’ convenience was very good in both treatment groups. The functional results of this study demonstrated statistically significant differences regarding the Harris Hip Score with higher values in the nail group compared with the blade plate group. This is in line with recent studies reporting that the Harris Hip Score demonstrated good results for both treatment concepts but indicating that cephalomedullary nailing was superior compared with extramedullary fixation methods [21,37].

However, it must be mentioned that complete radiological data were not available from all patients in the postoperative course. This could be due to the fact that patients who were satisfied with the healing process no longer presented to the clinic for follow-up, whereas more dissatisfied patients presented later in the course, which could have led to a bias in the long-term data. This could also apply to the SF-12 data. Especially in the assessment of the psychological component, but also in the summary of the physical component of the SF-12, it was not clear whether the responses could be attributed exclusively to the revision surgery or, for example, to existing preconditions or frailty due to older patient age or a combination of the aforementioned reasons.

### Study Limitations

A limitation of this study is first of all its retrospective nature. Therefore, it was not possible to randomize age, gender or indication for the treatment concept. Nevertheless, a strength might be seen in the relatively large cohort size, since this study deals with a rare entity which is difficult to find and to compare. While only a few cases are available in the literature, the results of this study with a long follow-up period of consecutive patients may be highly relevant. There was no effect on decision making initially due to the retrospective study design. The treatment indications were always considered on an individual patient basis. This included the assessment of the benefits and harms of every intervention. Finally, individual patient factors were considered in addition to the caput-collum-diaphyseal angle in the decision-making process. However, due to the retrospective nature, it remains not entirely clear how the weighting of these factors in individual cases influenced the decision to choose one procedure or the other.

## 5. Conclusions

This study demonstrated that the revision treatment of trochanteric and subtrochanteric nonunion using a 95-degree angled blade plate including the correction of varus malalignment or a cephalomedullary nail without varus correction results in a high percentage of bone union, with good clinical long-term outcomes and a low incidence of complications at the short-term and mid-term follow-up and for both methods. Therefore, based on the results of this study, both surgical revision concepts may be recommended to be adequate treatment options for these challenging situations. Treatment selection may be dependent on age, comorbidities and the degree of mobility prior to surgical revision. Due toshorter operation time and better functional long-term results, intramedullary nailing might be recommended, especially in geriatric patients. Further prospective studies with a larger patient population would be desirable.

## Figures and Tables

**Table 1:** Basic data overview.

Parameter	Blade Plate (n = 42)	CM Nail (n = 26)	*p*-Value
Age [years]	57 ± 13	58 ± 13	0.69
Gender [male/female]	28/14	19/7	0.58
Stay in hospital [days]	13 ± 7	11 ± 5	0.15
Duration of revision surgery [minutes]	202 ± 59	137 ± 47	<0.0001
AO/OTA classification [n] (31A1/31A2/31A3/32A1/32A2/32A3/32C2/32C3)	4/10/18/0/5/3/1/1	20/0/0/3/3/0/0/0	<0.0001

(n = number). Results are presented as mean ± standard deviation.

**Table 2:** Risk factors and preconditions.

Parameter [n]	Blade Plate	CM Nail	*p*-Value
Risk factors overall [yes/no]	39/3	25/1	0.57
BMI > 25	27/15	19/7	0.45
Nicotine abuse	16/26	7/19	0.34
Alcohol abuse	21/21	8/18	0.12
Drug abuse	1/41	1/25	0.73
Bisphosphonate	3/39	1/25	0.57
Cortisone	1/41	1/25	0.73
Non-steroidal anti-inflammatory drug (NSAID)	5/37	2/24	0.58
Preconditions overall [yes/no]	28/14	20/6	0.38
Diabetes mellitus	2/40	1/25	0.86
Coagulation disorder	2/40	2/24	0.62
Osteoporosis	5/37	3/23	0.96
Peripheral arterial disease	0/42	3/23	0.24
Chronic obstructive pulmonary disease (COPD)	5/37	1/25	0.26
Coronary heart disease	2/40	0/26	0.26
Hypertension	16/26	14/12	0.20
Heart disease	3/39	1/25	0.57
Rheumatoid Arthritis	1/41	0/26	0.43
Hypercholesterolemia	1/41	1/25	0.73
Morbus Perthes	2/42	0/26	0.26
Hip dysplasia	2/40	0/26	0.26
Polytrauma	2/20	4/22	0.13
Morbus Crohn	1/41	0/26	0.43
Breast cancer	2/40	1/25	0.86

(n = number).

**Table 3:** Short-term complications.

Parameter [n]	Blade Plate	CM Nail	*p*-Value
Overall complications (yes/no)	11/31	2/24	0.42
Blood transfusion	9/33	0/26	0.08
Local wound infection	2/40	0/26	0.26
Hematoma/Seroma	3/39	2/24	0.93

(n = number).

**Table 4:** Complications depending on length of 95-degree angled blade plate.

Length of Blade Plate	Short (n = 17)	Long (n = 25)	*p*-Value
Complications depending on length of blade plate [yes/no]	2/15	9/16	0.08

(n = number).

**Table 5:** Mid-term complication rates.

Parameter [n]	Blade Plate	CM Nail	*p*-Value
Complication rate (overall) [yes/no]	11/31	1/25	0.13
Surgical revision [yes/no]	11/31	1/25	0.06
Cut-out [yes/no]	0/31	1/25	0.20
Osseous union 12 months after surgical revision [yes/no]	24/10	22/1	0.018

(n = number).

**Table 6:** Overview of functional long-term results.

Parameter	Blade Plate (n = 27)	CM Nail (n = 16)	*p*-Value
HHS [points]	64 ± 23	81 ± 21	0.026
VAS—pain at rest [points]	2 ± 2	1 ± 2	0.47
VAS—pain on stress [points]	3 ± 2	2 ± 3	0.09
Physical component summary SF-12 [points]	40 (range 18–58)	45 (range 27–57)	0.33
Mental component summary SF-12 [points] (median)	46 (range 32–60)	53 (range 29–62)	0.08

(n = number). Results are presented as mean ± standard deviation.

## Data Availability

The dataset analyzed during the current work is available from the corresponding author upon reasonable request. Part of this dataset is content of an upcoming thesis of Annika Dolt.

## References

[1] Jaeschke-Melli S., Hedke J., Meiners J., Dannenberg O., Jürgens C., Faschingbauer M. (2013). Standards in the treatment of proximal femoral fractures. Trauma Berufskrankh.

[2] de Vries J.S., Kloen P., Borens O., Marti R.K., Helfet D.L. (2006). Treatment of subtrochanteric nonunions. Injury.

[3] Steinhausen E., Glombitza M., Böhm H.J., Hax P.M., Rixen D. (2013). Non-unions. From diagnosis to healing. Unfallchirurg.

[4] von Rüden C., Hungerer S., Augat P., Trapp O., Bühren V., Hierholzer C. (2015). Breakage of cephalomedullary nailing in operative treatment of trochanteric and subtrochanteric femoral fractures. Arch. Orthop. Trauma Surg..

[5] Grünewald D., Dolt A., Barzen S., Rehme-Röhrl J., von Rüden C., Hoffmann R., Schweigkofler U. (2024). Is the 95° blade plate still important in the treatment of proximal femoral pseudarthrosis?. Unfallchirurgie.

[6] Moldovan F. (2024). Sterile Inflammatory Response and Surgery-Related Trauma in Elderly Patients with Subtrochanteric Fractures. Biomedicines.

[7] Bano G., Dianin M., Biz C., Bedogni M., Alessi A., Bordignon A., Bizzotto M., Berizzi A., Ruggieri P., Manzato E. (2020). Efficacy of an interdisciplinary pathway in a first level trauma center orthopaedic unit: A prospective study of a cohort of elderly patients with hip fractures. Arch. Gerontol. Geriatr..

[8] Amorosa L.F., Jayaram P.R., Wellman D.S., Lorich D.G., Helfet D.L. (2014). The use of the 95-degree-angled blade plate in femoral nonunion surgery. Eur. J. Orthop. Surg. Traumatol..

[9] Swanson E.A., Garrard E.C., Bernstein D.T., O’Connor D.P., Brinker M.R. (2015). Results of a systematic approach to exchange nailing for the treatment of aseptic femoral nonunions. J. Orthop. Trauma.

[10] Schmidmaier G., Moghaddam A. (2015). Long bone nonunion. Z. Orthop. Unfall..

[11] Loizou C.L., McNamara I., Ahmed K., Pryor G.A., Parker M.J. (2010). Classification of subtrochanteric femoral fractures. Injury.

[12] Perren S.M., Fernandez A., Regazzoni P. (2015). Understanding fracture healing biomechanics based on the “strain” concept and its functional applications. Acta Chir. Orthop. Traumatol. Cech..

[13] Harris W.H. (1969). Traumatic arthritis of the hip after dislocation and acetabular fractures: Treatment by mold arthroplasty. An end-result study using a new method of result evaluation. J. Bone Joint Surg. Am..

[14] Ware J., Kosinski M., Keller S.D. (1996). A 12-Item Short-Form Health Survey: Construction of scales and preliminary tests of reliability and validity. Med. Care.

[15] Fisher J.S., Kazam J.J., Fufa D., Bartolotta R.J. (2019). Radiologic evaluation of fracture healing. Skeletal Radiol..

[16] Parker M.J., Handoll H.H. (2008). Gamma and other cephalocondylic intramedullary nails versus extramedullary implants for extra- capsular hip fractures in adults. Cochrane Database Syst. Rev..

[17] Wu S.C., Rau C.S., Kuo S.C.H., Chien P.C., Hsieh C.H. (2019). The influence of ageing on the incidence and site of trauma femoral fractures: A cross-sectional analysis. BMC Musculoskelet. Disord..

[18] Bhandari M., Schemitsch E., Jönsson A., Zlowodzki M., Haidukewych G.J. (2009). Gamma nails revisited: Gamma nails versus compression hip screws in the management of intertrochanteric fractures of the hip: A meta-analysis. J. Orthop. Trauma.

[19] Forward D.P., Doro C.J., OʼToole R.V., Kim H., Floyd J.C., Sciadini M.F., Turen C.H., Hsieh A.H., Nascone J.W. (2012). A biomechanical comparison of a locking plate, a nail, and a 95° angled blade plate for fixation of subtrochanteric femoral fractures. J. Orthop. Trauma.

[20] Benz D., Tarrant S.M., Balogh Z.J. (2020). Proximal femur fracture non-union with or without implant failure: A revision technique with clinical outcomes. Injury.

[21] Vicenti G., Solarino G., Bizzoca D., Simone F., Maccagnano G., Zavattini G., Ottaviani G., Carrozzo M., Buono C., Zaccari D. (2022). Use of the 95-degree angled blade plate with biological and mechanical augmentation to treat proximal femur non-unions: A case series. BMC Musculoskelet. Disord..

[22] Bhowmick K., Matthai T., Boopalan P.R.J., Jepegnanam T.S. (2020). Decision making in the management of malunion and non-union of intertrochanteric fractures of the hip. HIP Int..

[23] Dietze C., Brand A., Friederichs J., Stuby F., Schneidmueller D., von Rüden C. (2022). Results of revision intramedullary nailing with and without auxillary plate in aseptic trochanteric and subtrochanteric nonunion. Eur. J. Trauma Emerg. Surg..

[24] Rehme J., Woltmann A., Brand A., von Rüden C. (2021). Does auxiliary cerclage wiring provide intrinsic stability in cephalomedullary nailing of trochanteric and subtrochanteric fractures?. Int. Orthop..

[25] Eberle S., Bauer C., Gerber C., von Oldenburg G., Augat P. (2010). The stability of a hip fracture determines the fatigue of an intramedullary nail. Proc. Inst. Mech. Eng. H.

[26] Wiss D.A., Garlich J., Hashmi S., Neustein A. (2021). Risk Factors for Development of a Recalcitrant Femoral Nonunion: A Single Surgeon Experience in 122 Patients. J. Orthop. Trauma.

[27] Falkensammer M.L., Benninger E., Meier C. (2016). Reduction Techniques for Trochantericand Subtrochanteric Fractures of the Femur: A Practical Guide. Acta Chir. Orthop. Traumatol. Cech..

[28] Tetsunaga T., Fujiwara K., Endo H., Noda T., Tetsunaga T., Sato T., Shiota N., Ozaki T. (2017). Total hip arthroplasty after failed treatment of proximal femur fracture. Arch. Orthop. Trauma Surg..

[29] Hak D.J., Lee S.S., Goulet J.A. (2000). Success of exchange reamed intramedullary nailing for femoral shaft nonunion or delayed union. J. Orthop. Trauma.

[30] Shroeder J.E., Mosheiff R., Khoury A., Liebergall M., Weil Y.A. (2009). The outcome of closed, intramedullary exchange nailing with reamed insertion in the treatment of femoral shaft nonunions. J. Orthop. Trauma.

[31] Wu C.C. (2007). Exchange nailing for aseptic non-union of femoral shaft: A retrospective cohort study for efect of reaming size. J. Trauma Acute Care Surg..

[32] Barquet A., Mayora G., Fregeiro J., López L., Rienzi D., Francescoli L. (2004). The treatment of subtrochanteric nonunions with the long gamma nail: Twenty-six patients with a minimum 2-year follow-up. J. Orthop. Trauma.

[33] Haidukewych G.J., Berry D.J. (2004). Non-union of fractures of the subtrochanteric region of the femur. Clin. Orthop. Relat. Res..

[34] Lu Y., Uppal H.S. (2019). Hip Fractures: Relevant Anatomy, Classification, and Biomechanics of Fracture and Fixation. Geriatr. Orthop. Surg. Rehabil..

[35] Shukla S., Johnston P., Ahmad M.A., Wynn-Jones H., Patel A.D., Walton N.P. (2007). Outcome of traumatic subtrochanteric femoral fractures fxed using cephalo-medullary nails. Injury.

[36] Oestern H.J., Gänsslen A. (2010). The use of blade plate and dynamic screw plate osteosynthesis. Orthopade.

[37] Wang J., Li H., Jia H., Ma X. (2020). Intramedullary versus extramedullary fixation in the treatment of subtrochanteric femur fractures: A comprehensive systematic review and meta-analysis. Acta Orthop. Traumatol. Turc..

[38] Ravi B., Pincus D., Choi S., Jenkinson R., Wasserstein D.N., Redelmeier D.A. (2019). Association of Duration of Surgery with Postoperative Delirium Among Patients Receiving Hip Fracture Repair. JAMA Netw. Open.

